# Effects of Gualou Guizhi Decoction Aqueous Extract on Axonal Regeneration in Organotypic Cortical Slice Culture after Oxygen-Glucose Deprivation

**DOI:** 10.1155/2017/5170538

**Published:** 2017-09-18

**Authors:** Lihong Nan, Lan Yang, Yanfang Zheng, Yibo He, Qingqing Xie, Zheming Chen, Huang Li, Mei Huang

**Affiliations:** ^1^College of Pharmacy, Fujian University of Traditional Chinese Medicine, Fuzhou, Fujian 350122, China; ^2^Pharmaceutical Preparation Section, First Hospital of Fuzhou, Fuzhou, Fujian 350009, China

## Abstract

Gualou Guizhi decoction (GLGZD) is effective for the clinical treatment of limb spasms caused by ischemic stroke, but its underlying mechanism is unclear. Propidium iodide (PI) fluorescence staining, terminal deoxynucleotidyl transferase-mediated dUTP-biotin nick end labeling (TUNEL), immunohistochemistry, western blot, and real-time qPCR were used to observe the axonal regeneration and neuroprotective effects of GLGZD aqueous extract on organotypic cortical slices exposed to oxygen-glucose deprivation (OGD) and further elucidate the potential mechanisms. Compared with the OGD group, the GLGZD aqueous extract decreased the red PI fluorescence intensity; inhibited neuronal apoptosis; improved the growth of slice axons; upregulated the protein expression of tau and growth-associated protein-43; and decreased protein and mRNA expression of neurite outgrowth inhibitor protein-A (Nogo-A), Nogo receptor 1 (NgR1), ras homolog gene family A (RhoA), rho-associated coiled-coil-containing protein kinase (ROCK), and phosphorylation of collapsin response mediator protein 2 (CRMP2). Our study found that GLGZD had a strong neuroprotective effect on brain slices after OGD injury. GLGZD plays a vital role in promoting axonal remodeling and functional remodeling, which may be related to regulation of the expression of Nogo-A and its receptor NgR1, near the injured axons, inhibition of the Rho-ROCK pathway, and reduction of CRMP2 phosphorylation.

## 1. Introduction

Stroke, an acute cerebrovascular condition, is the second most common cause of disease-related death. Furthermore, limb dysfunction often occurs after stroke and is the most common postapoplectic sequela associated with the upper motor neuron injury caused by various factors during the ischemic process. A previous study showed that reduction of fat molecules, separation of the axonal medullary sheath, and edema in axons were eventually observed in the injured neurons [1]. Damage to the axonal structure is the leading cause and pathological basis for dysneuria and nerve diseases.

Various types of receptors exist on the surface of axonal growth cones. These receptors are highly sensitive to the surrounding microenvironment and identify specific signal factors in the extracellular matrix or other cells, ultimately affecting the orientation of axonal growth. The inhibition of the growth-inhibiting factors of the medullary sheath facilitates axonal regeneration and neural reorganization [2]. Therefore, regulation of the axonal microenvironment is critical to axonal regeneration [3].

Neurite outgrowth inhibitor protein-A (Nogo-A) is widespread in the mammalian central nervous system and is distributed in oligodendroglia and myelin. In physiological conditions, Nogo-A can stabilize the neural signaling pathways; however, it exerts an inhibitory effect on nerve growth and plasticity after neuronal injury [4]. Nogo-A causes growth cones to collapse and inhibits axon growth arrest by activating the small G protein ras homolog gene family member A (RhoA) and rho-associated coiled-coil protein kinase (ROCK) through Nogo receptor 1 (NgR1) in the RhoA/ROCK pathway [5, 6]. Several recent studies demonstrated that downregulation of Nogo-A expression or blockade of the Nogo-A pathway improves neural regeneration and the compensatory extension of nerve fibers compared with unaided regeneration by endogenous mechanisms alone [7].

Gualou Guizhi decoction (GLGZD) was first prescribed in the “Jinkui Yaolve” (a traditional Chinese medical work) by Zhongjing Zhang. It is composed of six Chinese herbs (*Trichosanthes kirilowii* Maxim.,* Paeonia lactiflora* Pall.,* Cinnamomum cassia* Presl.,* Glycyrrhiza uralensis* Fisch.,* Zingiber officinale* Rosc., and* Ziziphus jujuba* Mill.) in a ratio of 10 : 3 : 3 : 3 : 2 : 3 by weight, and it is effective in treating convulsive diseases. In traditional Chinese medicine, the category “convulsive disease” included back stiffness, tics of the limbs, and opisthotonos. Spasm and contraction of muscles and tendons were believed to be the common clinical characteristics [8]. Consequently, in its long history, GLGZD has primarily been used in China to treat postapoplectic limb spasm, spinal cord injury limb spasm, and epilepsy [9]. Clinical studies have found that GLGZD treatment has a beneficial effect on limb spasm after stroke [10], but its mechanism is unclear. Experimental studies by our research unit have shown that there are multiple mechanisms involved in the neuroprotective effect of GLGZD [11, 12].

The motor cortex is a highly evolved center that controls the execution of spontaneous bodily movements. Motor cortex injury induced by multiple mechanisms is the cause of disordered limb activity after stroke. The use of cortical brain slices has ushered in a new stage of research into the mechanisms of motor function rehabilitation [13, 14]. The central nervous system depends on a precise connection between the neuron and the target. The ability to reestablish the connections between neurons and denervated effector organs after stroke would greatly improve functional recovery.

At present, the effect of GLGZD on axonal regeneration has not been reported. In this study, we aimed to establish an oxygen-glucose deprivation (OGD) model via neonatal rat cortical slices to observe the neuroprotective and axonal regeneration effects of the GLGZD aqueous extract on organotypic cortical slices. In addition, we aimed to determine whether the GLGZD aqueous extract exerts its axonal regenerative effect through the Rho-ROCK signaling pathway mediated by the Nogo-A protein and its receptor to provide experimental evidence that GLGZD can improve the neurological function of stroke patients and promote the rehabilitation of neurological function after stroke.

## 2. Materials and Methods

### 2.1. Chemicals and Reagents

Gallic acid, oxypaeoniflorin, paeoniflorin sulfonate, albiflorin, paeoniflorin, liquiritin apioside, liquiritin, isoliquiritin apioside, isoliquiritin, liquiritigenin, cinnamic acid, cinnamaldehyde, isoliquiritigenin, glycyrrhizic acid, licochalcone A, and glycyrrhetinic acid were all purchased from the National Institute for the Control of Pharmaceutical and Biological Products (Beijing, China). Chromatographic grade formic acid, methanol, and acetonitrile were purchased from Merck Co. (Darmstadt, Germany). All other chemical reagents used in this research were of analytical grade.

### 2.2. Plant Material

The herbs were purchased from Tongchun Drugstore (Fuzhou, Fujian, China), and they were authenticated by Professor Yang (College of Pharmacy, Fujian University of Traditional Chinese Medicine). Voucher specimens were deposited in the College of Pharmacy of Fujian University of Traditional Chinese Medicine, Fujian, China.

### 2.3. Animals

Specific-pathogen-free (SPF) 7-to-9-day-old male Sprague-Dawley rats were obtained from the Laboratory Animal Center of Fujian University of Traditional Chinese Medicine (Fuzhou, China; license: SCXK (min) 2012-0001). The animals were maintained in the Laboratory Animal Center of Fujian University of Traditional Chinese Medicine (temperature at 22–25°C, relative humidity at 50%–70%). In the experiment, the mice were placed in a sterile laboratory for the preparation of brain slices. The present study was conducted in strict accordance with the recommendations in the Guide for the Care and Use of Laboratory Animals of the National Research Council [15]. The animal use protocol was reviewed and approved by the Institutional Animal Care and Use Committee of Fujian University of Traditional Chinese Medicine (Fuzhou, China).

### 2.4. Preparation of the GLGZD Aqueous Extract

The original prescription from the “Jinkui Yaolve” required the decoction of all medical materials by boiling in distilled water for 1 h, twice. The solution was then dried under a microwave dryer to obtain GLGZD aqueous extract powder. The GLGZD aqueous extract powder was then stored at −20°C for further analysis.

### 2.5. HPLC Analysis of the GLGZD Aqueous Extract

GLGZD aqueous extract powder was dissolved in 50% methanol, and the extract was filtered through 0.45 mm microporous membranes. The solutions were then injected into an HPLC system (Shimadzu, Kyoto, Japan) for analysis. A Shimadzu HPLC system coupled with a photodiode array detector was used to analyze the GLGZD. An LC-20A pump, a DGU-20A5 degasser, an SIL-20 Autoinjector, and a CTO-20A column thermostat were included in the HPLC system. Chromatographic separation was performed on a Dikma diamond C18 (ID 4.6 × 250 mm, 5 *μ*m), and the column temperature was maintained at 30°C. The mobile phase consisted of a 0.1% formic acid aqueous solution (A) and acetonitrile (B) with the following gradient elution program: 0–8 min, 5% B; 8–50 min, 5–35% B; 50–65 min, 35–50% B; 65–75 min, 50–60% B; 75–85 min, 60–70% B; and 85–100 min, 70–90% B. The flow rate and injection volume were 0.8 mL/min and 10 *μ*L, respectively. The analysis was performed in triplicate.

### 2.6. Preparation of Organotypic Cortical Slice Culture

The methods developed by Stoppini et al., Dong and Buonomano, and Foehring et al. were applied [16–18]. The skin of the neonatal rats was sterilized with 75% medicinal alcohol before decapitation. The brains were obtained and immersed in ice-cold minimal essential medium (MEM, HyClone, Logan, UT, USA). The cerebellums and brainstems were removed. The brains were divided into two halves along the sagittal plane with a scalpel and adhered to a buffer tray in a tissue vibratome (Campden Instrument, Loughborough, UK) with the frontal poles upward. Slices of 400 *μ*m thickness were cut along the coronal plane with a vibratome. The cortical sections were separated under a stereomicroscope and immediately transferred to semiporous cellulose membrane inserts (Millicell-CM, 0.4 *μ*m, Millipore, Boston, MA, USA). Each insert contained three cortical slices. The inserts were placed in six-well plates with 1 mL of culture medium per well. The six-well plates were then placed in an incubator (Thermo Scientific, Waltham, MA, USA) at 37°C with a 5% CO_2_ and 95% O_2_. The culture medium was replaced after 2 d and subsequently every 3 d. The composition of the culture medium was as follows: 25% Hank's balanced salt solutions (HBSS, HyClone, Logan, UT, USA) + 50% MEM + 25% horse serum (Gibco, Carlsbad, CA, USA) + 6.5 g/L D-glucose (Sigma, St. Louis, MO, USA) + 25 mM 4-(2-hydroxyethyl)-1-piperazineethanesulfonic acid buffer (HEPES, Sigma, St. Louis, MO, USA), pH 7.2–7.4, + 100 U/mL of two antibiotics.

### 2.7. Establishment of an OGD Injury Model

After 14 d of culture, all slices with uniform thickness and good transmittance were selected. The slices were washed three times in PBS, and 1 mL of PBS was added to each well. They were then cultured in an incubator at 37°C with 94% N_2_, 5% CO_2_, and 1% O_2_ for 45 min to establish the OGD model.

### 2.8. Experimental Grouping and Treatment

Twenty milligrams of GLGZD aqueous extract powder was dissolved in 2 mL aseptic PBS to produce a 10 mg/mL stock solution, which was then filtrated through 0.22 mm microporous membranes and cold-preserved at −20°C. Stock solution was diluted to the desired concentration with culture medium before use. After the slices were cultured for 14 d, they were divided into five groups: (1) control group: maintained under normal conditions; (2) OGD group: the PBS was replaced with 1 mL culture medium in the hole, followed by OGD for 45 min; (3) GLGZD aqueous extract low-dose group (GLGZD-L): OGD for 45 min, followed by replacing the PBS with 1 mL culture medium containing 50 *μ*g/mL GLGZD aqueous extract; (4) GLGZD aqueous extract middle-dose group (GLGZD-M): OGD for 45 min, followed by replacing the PBS with 1 mL culture medium containing 100 *μ*g/mL GLGZD aqueous extract; and (5) GLGZD aqueous extract high-dose group (GLGZD-H): OGD for 45 min, followed by replacing the PBS with 1 mL culture medium containing 200 *μ*g/mL GLGZD aqueous extract. Each group was assigned three wells, with three slices for each hole. All slices were cultured in a CO_2_ incubator for 3 d and then collected for further studies.

### 2.9. Propidium Iodide (PI) Staining

After the cultures had been treated with the GLGZD aqueous extract for 3 d, the culture medium was replaced with 1 mL of 50 *μ*g/mL PI (Sigma, St. Louis, MO, USA) per well. They were further cultured in an incubator at 37°C with 5% CO_2_ and 95% O_2_ for 1 h; cell damage was then observed using a fluorescence microscope (Leica, Wentzler, Germany). ImageJ was employed to analyze the IOD.

### 2.10. Terminal Deoxynucleotidyl Transferase-Mediated dUTP-Biotin Nick End Labeling Assay (TUNEL)

After measurement of GLGZD aqueous extract for 3 d, the slices were fixed with 4% paraformaldehyde for 15 min. All slices were washed with PBS twice, 5 min/wash, and were consequently incubated with 2% Trion X-100 for permeabilizing at 4°C overnight. After fixation with 4% paraformaldehyde for another 5 min, the brain slices were cut together with the microporous membrane and placed on a clean slide. Next, 100 *μ*L of the equilibrium buffer was added, completely covering the slices for 10 min incubation at room temperature. The equilibrium buffer was carefully removed with absorbent paper; 50 *μ*L terminal deoxynucleotidyl transferase (TdT) reaction liquid, including the equilibrium solution and FITC-dUTP as well as TdT enzyme (Promega, Madison, WI, USA) was added in the dark. Parafilm was employed to cover the slide. The slices were incubated in a 37°C incubation chamber for 60 min in the dark. After removing the parafilm, the tissues were soaked in the SSC for 15 min to terminate the reaction, and 100 *μ*L DAPI (Beyotime Biotechnology, Beijing, China) was added for 10 min. Finally, antiquenching agent was used to mount and protect the samples from light. The procedure described above should be performed in the dark. Laser confocal microscopy (CARL ZEISS, Oberkochen, Germany) was used to observe the apoptotic cells. After TUNEL staining, apoptotic cells showed green fluorescence. ImageJ software was used to calculate the average number of TUNEL-positive cells in 5 sections of each slice; apoptosis rate (%) = apoptotic cells/total cell number × 100%.

### 2.11. Immunohistochemistry (IHC)

After 3 d of treatment with the GLGZD aqueous extract, the brain slices of each group were removed from the incubator, fixed in 4% paraformaldehyde, and dehydrated in 30% sucrose overnight. The dehydrated brain slices were cut together with the microporous membrane, and the tissue was adhered on the surface of the frozen stage. Serial slices were obtained at a thickness of 10 *μ*m and stored at −20°C. The slices were immersed in a sodium citrate buffer solution (Solarbio, Beijing, China) and boiled for antigen retrieval. The endogenous peroxidase was blocked, followed by the nonspecific binding sites. A primary antibody against neurofilament 68 (NF68) (1 : 400, Novusbio, Littleton, CO, USA) was applied to the slices overnight at 4°C. PBS was applied to the negative control group in place of the antibody. The slices were incubated with goat anti-rabbit antibody (Maixin Biotechnology Development Co., Ltd., Fuzhou, China) and then incubated with* Streptomyces* avidin labeled with horseradish peroxidase (HRP, Maixin Biotechnology Development Co. Ltd., Fuzhou, China). The slices were stained with 3,3′-diaminobenzidine (DAB) and counterstained with hematoxylin. An inverted fluorescence microscope (Leica, Wetzlar, Germany) was used to observe and record the immunofluorescence. Five fields of the cortical motor area in each slice were selected with a high-power lens. The lengths of ten random positive axons in each field were measured and averaged using ImageJ software. The mean lengths were calculated from the measured values of the lengths of the axons in each slice.

### 2.12. Western Blot

The slices were washed with PBS, after which 400 *μ*L RIPA lysis buffer was added to each well to lyse the cells. The brain slices were scraped off the microporous membrane with a cell scraper and transferred, together with the lysate, to a 1.5 mL centrifuge tube. The cells were homogenized and lysed for 30 min after centrifugation using an ultrasonic homogenizer. The lysate was centrifuged (Eppendorf, Hamburg, Germany) at 13,000 rpm at 4°C to acquire the supernatant. A bicinchoninic acid (BCA) assay was used to measure the protein concentration. The proteins were denatured by boiling for 5 min. Equal amounts of protein (50 *μ*g) were separated by 10% sodium dodecyl sulfate-polyacrylamide gel electrophoresis (SDS-PAGE) and then transferred to nitrocellulose (NC) membranes using electricity. The transferred NC membrane was treated with 5% BSA (bovine serum albumin, phosphorylated protein) or 5% skim milk (nonphosphorylated protein) for 2 h to block the nonspecific binding sites. The following antibodies were added: mouse anti-rat tau monoclonal antibody (1 : 1000, Millipore, Boston, MA, USA); rabbit anti-rat growth-associated protein 43 (GAP43) polyclonal antibody (1 : 1000, Sigma, St. Louis, MO, USA); goat anti-rat Nogo-A polyclonal antibody (1 : 1000, Novusbio, Littleton, CO, USA); rabbit anti-rat NgR1 polyclonal antibody (1 : 1000, Abcam, Cambridge, UK); rabbit anti-rat RhoA monoclonal antibody (1 : 1000, Cell Signaling Technology, Boston, MA, USA); rabbit anti-rat ROCK monoclonal antibody (1 : 1000, Cell Signaling Technology); rabbit anti-rat collapsin response mediator protein 2 (CRMP2) monoclonal antibody (1 : 1000, Cell Signaling Technology, Boston, MA, USA); rabbit anti-rat pCRMP2 monoclonal antibody (1 : 1000, Cell Signaling Technology). The membranes were incubated overnight at 4°C. HRP-conjugated rabbit anti-goat IgG (1 : 3000, Vazyme Biotech Co., Ltd., Nanjing, China) was added, and the membrane was incubated for 2 h. The membrane was then imaged in a gel imaging system (Bio-Rad, Hercules, CA, USA) with electrochemiluminescence (ECL). ImageJ was used for quantitative analysis of the gray value of each band. *β*-Actin was used as the internal control. Three replicates of the experiments were performed.

### 2.13. Real-Time Quantitative Polymerase Chain Reaction (qPCR) Analysis

The slices and inserters were washed with PBS, and 1 mL Biozol Reagent (Biomiga Inc., San Diego, CA, USA) was added to each well of the inserters. Total RNA was extracted according to the manufacturer's instructions. For cDNA reverse transcription, a first strand cDNA synthesis kit was used. The cDNA was subjected to qPCR assays with the ChamQ SYBR qPCR master mix (Vazyme Biotech Co., Ltd.). The specific primers are as follows: Nogo-A, 5′-TCCAGTGTATCTAAGGCATCCAT-3′ (forward) and 5′-CAGAGACAGCAGCAGGAATAAG-3′ (reverse); NgR1, 5′-CCGACAACACCTTCCGAGAC-3′ (forward) and 5′-AGGAGACGGTCAAGACTGTGC-3′ (reverse); RhoA, 5′-ACTGGTGATTGTTGGTGATGG-3′ (forward) and 5′-GTCCACTTCAATATCTGCCACAT-3′ (reverse); ROCK, 5′-CAGAAGCAGTTAGAAGAAGCGAAT-3′ (forward) and 5′-AGGTCTCCAATCATCTCAGAATCA-3′ (reverse); GAPDH, 5′-CAACGGGAAACCCATCACCA-3′ (forward) and 5′-ACGCCAGTAGACTCCACGACAT-3′ (reverse).

The PCR detection procedure was as follows: denaturation for 30 s at 95°C, 10-s hold at 95°C, and 30-s hold at 60°C, followed by 40 cycles of stage 2. The solubility curve could be acquired in stage 3, which consisted of a 15-s hold at 95°C, 60-s hold at 60°C, and 15-s hold at 95°C. The comparative cycle threshold (CT) method was applied to obtain the expression levels of the target genes, and GADPH was used as the internal control to normalize the other values. All experiments were performed in triplicate.

### 2.14. Statistical Analysis

The data are expressed as the mean ± SE. One-way analysis of variance (ANOVA) was performed with SPSS 22.0 statistical software (Chicago, IL, USA). The least significant difference test was to evaluate multiple group differences when the data followed a normal distribution with an equal variance. The Game-Howell test was used when the data fulfilled the normal distribution but the variance was not equal. Nonparametric tests were used to evaluate multiple group differences if the data did not follow the normal distribution. *p* < 0.05 was considered statistically significant.

## 3. Results

### 3.1. HPLC Analysis of the GLGZD Aqueous Extract

**Figure 1:**
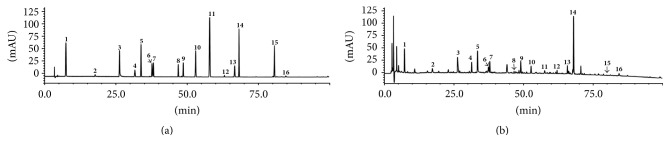
Chromatograms of the GLGZD aqueous extract and sixteen reference compounds. (a) HPLC-UV chromatograms of sixteen standard reference compounds at 254 nm. (b) HPLC-UV chromatograms of GLGZD aqueous extract monitored at 254 nm. (1) Gallic acid, (2) oxypaeoniflorin, (3) paeoniflorin sulfonate, (4) albiflorin, (5) paeoniflorin, (6) liquiritin apioside, (7) liquiritin, (8) isoliquiritin apioside, (9) isoliquiritin, (10) liquiritigenin, (11) cinnamic acid, (12) cinnamaldehyde, (13) isoliquiritigenin, (14) glycyrrhizic acid, (15) licochalcone A, and (16) glycyrrhetinic acid.

The major components of the GLGZD aqueous extract were analyzed by HPLC (Figure 1). Sixteen compounds were identified by comparison with standard reference compounds: gallic acid (1), oxypaeoniflorin (2), paeoniflorin sulfonate (3), albiflorin (4), paeoniflorin (5), liquiritin apioside (6), liquiritin (7), isoliquiritin apioside (8), isoliquiritin (9), liquiritigenin (10), cinnamic acid (11), cinnamaldehyde (12), isoliquiritigenin (13), glycyrrhizic acid (14), licochalcone A (15), and glycyrrhetinic acid (16). The percentage content of the sixteen compounds was estimated by a calibration curve method, yielding the following composition: gallic acid (0.2851 mg/g), oxypaeoniflorin (0.0266 mg/g), paeoniflorin sulfonate (2.4856 mg/g), albiflorin (1.7868 mg/g), paeoniflorin (2.789 mg/g), liquiritin apioside (0.1223 mg/g), liquiritin (0.3425 mg/g), isoliquiritin apioside (0.0158 mg/g), isoliquiritin (0.1931 mg/g), liquiritigenin (0.1026 mg/g), cinnamic acid (0.0316 mg/g), cinnamaldehyde (0.0174 mg/g), isoliquiritigenin (0.1498 mg/g), glycyrrhizic acid (4.7934 mg/g), licochalcone A (0.0139 mg/g), and glycyrrhetinic acid (0.0188 mg/g).

### 3.2. Effect of the GLGZD Aqueous Extract on PI Fluorescence Intensity

**Figure 2:**
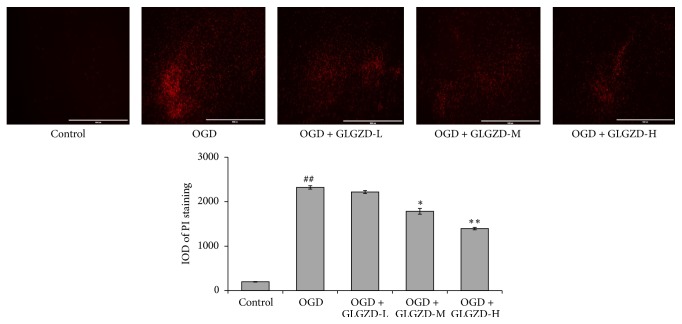
The effects of the GLGZD aqueous extract on PI fluorescence intensity in neonatal rat cortical slices. Fluorescence staining was applied to neonatal rat cortical slices (OGD for 45 min) after treatment with 50, 100, and 200 *μ*g/mL of GLGZD aqueous extract per day for 3 d. All data are expressed as the mean ± SE. ^##^*p* < 0.01 versus the control group; ^*∗*^*p* < 0.05, ^*∗∗*^*p* < 0.01 versus the OGD group.

The following results are presented in Figure 2. There is no observable PI fluorescence intensity (red) in the control group. The integrated optical density (IOD) value of the OGD group was markedly increased compared with that of the control group (*p* < 0.01) and was markedly decreased after treatment with either 100 or 200 *μ*g/mL of the GLGZD aqueous extract for 3 d compared with that of the control group (*p* < 0.05 or *p* < 0.01).

### 3.3. Effect of GLGZD Aqueous Extract on Cell Apoptosis

**Figure 3:**
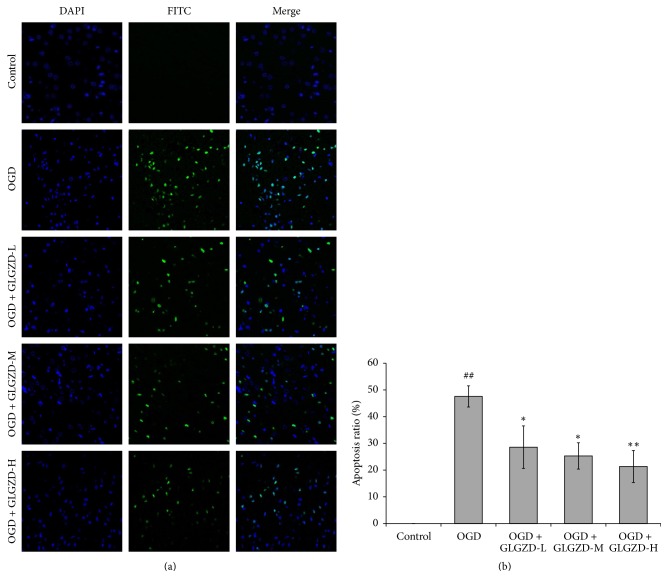
The effect of GLGZD aqueous extract on cell apoptosis in neonatal rat cortical slices. After DAPI staining, the nuclei showed blue fluorescence, and TUNEL labeling of apoptotic cells showed green fluorescence. Thus, the number of cell apoptosis is expressed as FITC in the figure, and DAPI indicates the total number of cells. The apoptosis rate was calculated by the following formula: apoptosis rate (%) = apoptotic cells/total number of cells × 100%. All data are expressed as the mean ± SE. ^##^*p* < 0.01 versus the control group; ^*∗*^*p* < 0.05, ^*∗∗*^*p* < 0.01 versus the OGD group.

The result of cell apoptosis is presented in Figure 3. Compared with the control group, the OGD group showed significantly increased cell apoptosis (*p* < 0.01). The number of apoptotic cells in GLGZD aqueous extract groups decreased compared with the OGD group, indicating that the apoptosis rate of the cells decreased (*p* < 0.05, *p* < 0.01).

### 3.4. Effect of the GLGZD Aqueous Extract on Axonal Regeneration

**Figure 4:**
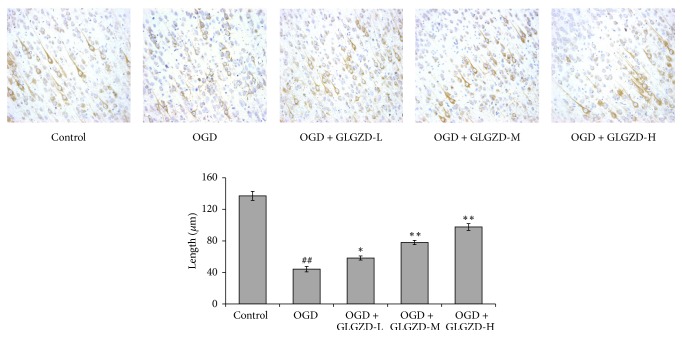
The effects of the GLGZD aqueous extract on axonal morphology and length after OGD in neonatal rat cortical slices. Immunohistochemical methods were used to label NF68 protein in brain slices, and the morphology and axon length were observed. Five fields of the cortical motor area in each slice were selected with a high-power lens. The lengths of ten random positive axons in each field were measured and averaged using ImageJ software. The mean lengths were calculated from the measured lengths of the axons in each slice. All data are expressed as the mean ± SE. ^##^*p* < 0.01 versus the control group; ^*∗*^*p* < 0.05, ^*∗∗*^*p* < 0.01 versus the OGD group.

The axonal regeneration results are presented in Figure 4. Morphology and manifest extension of the axons were observed in the control group. The axonal lengths in the OGD group were significantly less than those of the control group (*p* < 0.01). In the OGD group, the morphological integrity of the axons was disrupted, and many of the axonal fibers were fragmented. Axonal growth was markedly improved, and the lengths of the axons were increased in the GLGZD-L (50 *μ*g/mL), GLGZD-M (100 *μ*g/mL), and GLGZD-H (200 *μ*g/mL) groups in a dose-dependent manner compared with the ODG group (*p* < 0.05, *p* < 0.01).

### 3.5. Effects of the GLGZD Aqueous Extract on Tau, GAP43, Nogo-A, NgR1, RhoA, ROCK, CRMP2, and p-CRMP2 Expression

**Figure 5:**
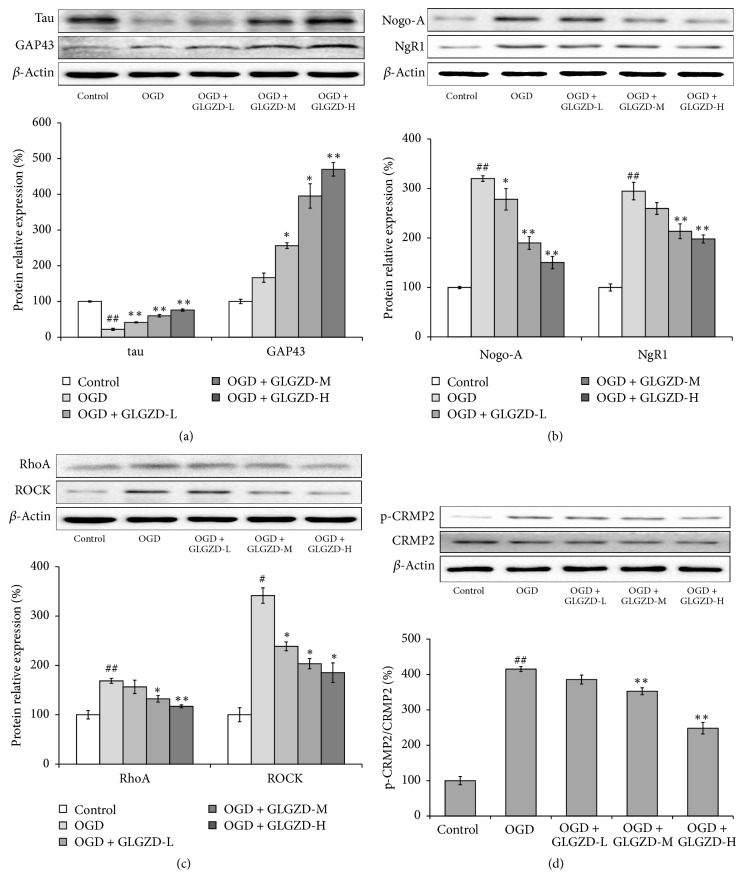
The effects of the GLGZD aqueous extract on protein expression in OGD neonatal rat cortical slices. The protein expression levels of* tau, GAP43*, Nogo-A, NgR1, RhoA, ROCK, p-CRMP2, and CRMP2 were measured by western blot. *β*-Actin was used as the internal control. All data are expressed as the mean ± SE. ^#^*p* < 0.05, ^##^*p* < 0.01 versus the control group; ^*∗*^*p* < 0.05, ^*∗∗*^*p* < 0.01 versus the OGD group.

The protein expression levels of* tau, GAP43*, Nogo-A, NgR1, RhoA, ROCK, CRMP2, and p-CRMP2 were examined by western blot (Figure 5). In the control group, the expression of tau protein in brain slices was high, but the expression of GAP43 protein was low. After 45 min of OGD, tau protein expression was decreased compared with that of the control group (*p* < 0.01), and there was no statistically significant difference in GAP43 protein expression; however, the GAP43 protein expression was upregulated compared with that of the control group (*p* > 0.05). In the GLGZD-L (50 *μ*g/mL), GLGZD-M (100 *μ*g/mL), and GLGZD-H (200 *μ*g/mL) groups, the expression levels of both tau and GAP43 protein were markedly upregulated compared with the levels in the OGD group (*p* < 0.05 or *p* < 0.01), in a dose-dependent manner. OGD prominently increased the expression of Nogo-A, NgR1, RhoA, and ROCK and the phosphorylation levels of p-CRMP2 compared with the levels in the control group (*p* < 0.05 or *p* < 0.01); however, these effects were mitigated in the GLGZD-M (100 *μ*g/mL) and GLGZD-H (200 *μ*g/mL) groups (*p* < 0.05 or *p* < 0.01) in a dose-dependent manner.

### 3.6. Effects of the GLGZD Aqueous Extract on the mRNA Expression of Nogo-A, NgR1, RhoA, and ROCK

**Figure 6:**
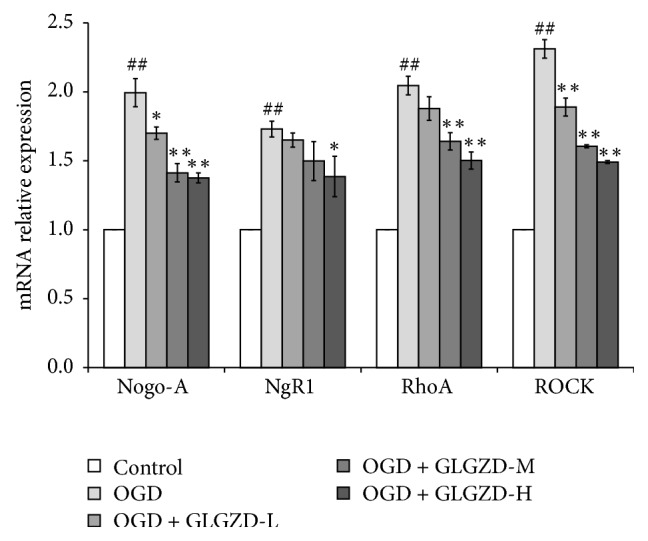
The effect of the GLGZD aqueous extract on mRNA expression in OGD neonatal rat cortical slices. qPCR was used to detect the mRNA expression of Nogo-A, NgR1, RhoA, and ROCK. GAPDH was used as the internal control. All data are expressed as the mean ± SE. ^##^*p* < 0.01 versus the control group; ^*∗*^*p* < 0.05, ^*∗∗*^*p* < 0.01 versus the OGD group.

The mRNA expression levels of Nogo-A, NgR1, RhoA, and ROCK were detected by qPCR (Figure 6). The mRNA expression levels of NgR1, RhoA, Nogo-A, and ROCK in the OGD group were significantly upregulated compared with the levels in the control group (*p* < 0.01). The mRNA expression levels of Nogo-A, NgR1, RhoA, and ROCK were significantly decreased after treatment with the GLGZD aqueous extract in the high-dose (200 *μ*g/mL) group compared with the levels in the OGD group (*p* < 0.05, *p* < 0.01).

## 4. Discussion

Organotypic brain slice culture, established by Stoppini et al. [16], is a promising technique to avoid the effects of the blood-brain barrier, ion concentration, temperature, pH, and other individual differences and to maintain the structure of the cell and the complete neural circuits to simulate the physiological environment* in vivo*. In organotypic culture, one can effectively monitor the microenvironment around specific cells. The choice of brain tissue donors is crucial to the development of organotypic brain slices [19]. Compared with mature individuals, younger animals have better morphology, a higher survival rate after transplantation, and greater vulnerability to damage in a damage model [20]. The success rate of organotypic brain slices culture was shown to decrease significantly as the donor rats matured [21]. Therefore, we chose 7–9-day-old neonatal rats to obtain the cortical slices.

The OGD model can be used to simulate the OGD environment observed in the process of ischemic stroke, and there are well-established procedures for using organotypic brain slice preparations as an OGD model [22, 23]. PI, used for nuclear staining, passes through disrupted cell membranes to combine with DNA and can be detected by its bright red fluorescence. In living cells, PI does not permeate the membrane. Therefore, it is generally used to detect the integrity and cytoactivity of nerve cells [24]. Studies have shown that apoptosis is the major form of injury in the cerebral ischemic area after cerebral ischemia reperfusion injury. This is closely related to the occurrence and development of cerebral infarction [25]. The apoptosis of ischemic neurons is delayed and reversible, thus providing time for the treatment of cerebral injury [26, 27]. In our study, there was no observable red fluorescence in the control group, indicating that the activity of the cortical brain slices was normal. The red fluorescence of the OGD group was significantly higher than that of the control (*p* < 0.01). Moreover, TUNEL staining showed that the apoptosis of cortical slices cells in the OGD group was significantly higher than in the control group (*p* < 0.01). These results indicated that OGD may cause the neuronal damage and cell apoptosis in cortical brain slices, which indicates that the model was successful. After treatment with the GLGZD aqueous extract for 3 d, the red fluorescence decreased significantly, and thus, cell apoptosis significantly decreased (*p* < 0.05 or *p* < 0.01). The above results clearly indicate that GLGZD can substantially improve the neuronal damage of brain slices after OGD and inhibit the apoptosis of nerve cells and thus provides effective neuroprotection. This result is in agreement with the findings of previous studies [11, 12].

Axons play an important role in transmitting signals to other neurons, muscular tissues, and glands. The generation of a new axon begins with the growth cone located on the axon terminal. During the ischemic process, the cytoskeletons of damaged axons collapse, and substance transport and signal transmission cease [28]. In the later period of axonal injury, axonal regeneration occurs and new functional connections are built [29].

Intermediate filaments, tubulin, and microfilaments form the cytoskeleton. Neurofilaments in the CNS are important for axonal generation and axonal diameter maintenance. A neurofilament (NF) is a protein triplet consisting of NF68, NF160, and NF200 [30, 31], which are primarily expressed in motoneurons. Therefore, immunologically labeled neurofilaments can be used to visualize the axons in brain tissues [32]. This study labeled NF68 by immunohistochemistry, revealing intact axons in the control group and heavily fragmented axonal fibers in the OGD group. The length along the axis was markedly shorter in the OGD group than in the control group (*p* < 0.01). This result suggests that the damage to the cortical slices after 45 min of OGD directly led to the collapse of the axonal structure. The GLGZD aqueous extract intervention significantly improved the shape of the axons, and the extension distance of the axons increased as the GLGZD aqueous extract dose increased (*p* < 0.05 or *p* < 0.01).

Tau is a microtubule-associated protein, and it is primarily expressed in axons. The aggregation of tau and tubulin stabilizes the axonal structure and facilitates tubulin assembly and axoplasmic transport [33]. Dysfunctional tau disorganizes the cytoskeleton under pathological conditions, hindering synaptic transmission and nutrient transport [34]. Therefore, tau levels indirectly reflect axonal growth. Our results demonstrated that tau expression significantly reduced after 45 min of OGD (*p* < 0.01), which suggests severe damage to the tubulins in the axons and blockade of axoplasmic transport. The GLGZD aqueous extract treatment markedly upregulated the expression of tau (*p* < 0.01), indicating that this intervention helped maintain the morphology of the axons.

GAP43 is a marker that plays an important role in axonal growth and synaptic plasticity [35]. Some studies have demonstrated that the body can initiate a certain degree of repair to promote axonal regeneration and reconstruction after stroke [36]. Our results demonstrate that GAP43 expression was lower in the control group and upregulated in the OGD group, which suggests that this upregulation was related to the recovery mechanism mentioned above. After treatment with the GLGZD aqueous extract, a further increase in GAP43 expression was observed (*p* < 0.05 or *p* < 0.01), suggesting that the GLGZD aqueous extract amplified the self-recovery process.

Studies have shown that the microenvironment of neurons determines their capacity for nerve growth. It has been demonstrated that Nogo-A can inhibit axonal regeneration after CNS damage. This effect can be blocked by a Nogo-A inhibitor, which markedly enhances neuroplasticity and neuronal functions [37, 38]. The Nogo-A-mediated Rho-ROCK pathway is involved in many important aspects of neural functions, including nerve growth and retraction. It has an important influence on the process of injury repair after stroke [39]. Rho protein has GTPase enzyme activity and can convert between GDP- and GTP-bound forms. Rho plays the role of a molecular switch in the regulation of intracellular signaling pathways. Nogo-A can activate the guanine nucleotide exchange factors (GEFs) by acting on the NgR1 receptor, which induces the transformation of Rho from inactive Rho-GDP to active Rho-GTP. RhoA is a small molecular weight G protein in the Rho family. Active RhoA reacts with one of its downstream substrates, ROCK, and binds to its coiled-coil domain alpha, which activates ROCK and the intracellular cascade [40]. CRMP2 is a major substrate of ROCK and plays an important role in the growth of axons and in stabilizing polymeric tubulin [41, 42]. CRMP2 can be phosphorylated by various neuronal proteins, which eliminates its ability to bind with tubulin heterodimers. The stability of the tubulins declines, and the growth cone collapses, disturbing axonal regeneration [43]. Our results demonstrated that both the protein and mRNA expression of RhoA, ROCK, Nogo-A, and its receptor NgR1 in cortical slices increased significantly after OGD and that the phosphorylation level of CRMP2 was also increased (*p* < 0.05 or *p* < 0.01). Our findings suggest that the Rho-ROCK pathway was activated by Nogo-A after OGD, thereby decreasing microtubule stability and inhibiting axonal regeneration in the brain issue. The GLGZD aqueous extract treatment for 3 d reduced the protein and mRNA expression levels of Nogo-A and its receptor NgR1 compared with the levels in the OGD group (*p* < 0.05 or *p* < 0.01). The protein and mRNA expression levels of RhoA and ROCK, which are two Rho-ROCK signaling pathway relative proteins, were downregulated to a certain degree, and the phosphorylation of CRMP2 was also decreased (*p* < 0.05 or *p* < 0.01).

The compounds in the GLGZD aqueous extract were qualitatively analyzed by HPLC to study the quality control of the product and identify potential pharmacodynamical substances. In our study, 16 compounds were identified by comparison to standard substances. We found that paeoniflorin, glycyrrhizin, and glycyrrhizic acid were more abundant than the other compounds in the GLGZD aqueous extract. Furthermore, studies have shown that these components can penetrate the blood-brain barrier after cerebral ischemia reperfusion injury and can thus alleviate the brain injury after cerebral ischemia and have a neuroprotective effect [44]. Therefore, it is likely that these compounds are the effective constituents that promote morphological remodeling and functional reconstruction after OGD. The identification of these active constituents lays a solid foundation for further research on the quality control and pharmacodynamic materials of GLGZD.

## 5. Conclusion

In conclusion, the GLGZD aqueous extract had a strong neuroprotective effect on brain slices after OGD injury. The GLGZD aqueous extract plays a vital role in promoting axonal remodeling and functional remodeling, which may be related to regulation of the expression of Nogo-A and its receptor NgR1 near the injured axons, inhibition of the Rho-ROCK pathway, and reduction of CRMP2 phosphorylation. Our study suggests that the GLGZD aqueous extract can improve the microenvironment of injured axons and inhibit restrictive factors to promote axonal regeneration. This study provides new ideas and a new research platform for the neuroprotective mechanism of GLGZD in ischemic stroke.
